# Choosing and changing the analysis scale in non-inferiority trials with a binary outcome

**DOI:** 10.1177/17407745211053790

**Published:** 2021-10-24

**Authors:** Zhong Li, Matteo Quartagno, Stefan Böhringer, Nan van Geloven

**Affiliations:** 1 Leiden Institute of Advanced Computer Science (LIACS), Leiden University, Leiden, The Netherlands; 2 Institute for Clinical Trials and Methodology, University College London, London, UK; 3 Department of Biomedical Data Sciences, Section Medical Statistics, Leiden University Medical Center, Leiden, The Netherlands

**Keywords:** Non-inferiority trial, non-inferiority margin, risk difference, risk ratio, odds ratio, sample size calculation

## Abstract

**Background:**

The size of the margin strongly influences the required sample size in non-inferiority and equivalence trials. What is sometimes ignored, however, is that for trials with binary outcomes, the scale of the margin – risk difference, risk ratio or odds ratio – also has a large impact on power and thus on sample size requirement. When considering several scales at the design stage of a trial, these sample size consequences should be taken into account. Sometimes, changing the scale may be needed at a later stage of a trial, for example, when the event proportion in the control arm turns out different from expected. Also after completion of a trial, a switch to another scale is sometimes made, for example, when using a regression model in a secondary analysis or when combining study results in a meta-analysis that requires unifying scales. The exact consequences of such switches are currently unknown.

**Methods and Results:**

This article first outlines sample size consequences for different choices of analysis scale at the design stage of a trial. We add a new result on sample size requirement comparing the risk difference scale with the risk ratio scale. Then, we study two different approaches to changing the analysis scale after the trial has commenced: (1) mapping the original non-inferiority margin using the event proportion in the control arm that was anticipated at the design stage or (2) mapping the original non-inferiority margin using the observed event proportion in the control arm. We use simulations to illustrate consequences on type I and type II error rates. Methods are illustrated on the INES trial, a non-inferiority trial that compared single birth rates in subfertile couples after different fertility treatments. Our results demonstrate large differences in required sample size when choosing between risk difference, risk ratio and odds ratio scales at the design stage of non-inferiority trials. In some cases, the sample size requirement is twice as large on one scale compared with another. Changing the scale after commencing the trial using anticipated proportions mainly impacts type II error rate, whereas switching using observed proportions is not advised due to not maintaining type I error rate. Differences were more pronounced with larger margins.

**Conclusions:**

Trialists should be aware that the analysis scale can have large impact on type I and type II error rates in non-inferiority trials.

## Introduction

For ethical reasons, in several disease areas it is becoming increasingly difficult to justify testing the efficacy of new treatments against placebo. Instead, active controlled trials are being used to test whether a new treatment which may be cheaper, safer, less invasive or easier to use has no worse efficacy than an already known effective treatment.^1^ No worse efficacy is defined as the difference between the new and the known effective treatment being bounded by a pre-specified margin that is considered clinically unacceptable.^2^ As pointed out by Mauri and D’Agostino,^3^ the use of such non-inferiority trials has increased considerably over the last decades.

Choosing the non-inferiority margin, which defines what we consider ‘not unacceptably worse’, is a pivotal step in designing non-inferiority trials. It is well known that the size of the margin strongly influences the required sample size. What is sometimes ignored, however, is that the scale of the margin – for binary endpoints risk difference (RD), risk ratio (RR) or odds ratio (OR) – also has a strong impact on the power of the trial and thus on the required sample size. Under seemingly equal assumptions, different scales for the analysis and corresponding non-inferiority margin may lead to different sample size requirements. Although this phenomenon has been pointed out in some statistical papers,^4–6^ it is not known to many trialists. Online tools or software packages for sample size calculation sometimes fail to offer the option of specifying the non-inferiority hypothesis on all three scales, in such instances typically only facilitating input on the RD scale. No comprehensive overview exists in which all three scales are compared for different design settings. The aim of this article is to provide such an overview.

Considering different analysis scales is common and recommended practice at the design stage of a trial. However, even after the trial has commenced, there may be unforeseen situations that warrant reconsidering the scale. In the first place, when the observed risk in the control arm turns out different from expected, for example during a blinded review of the data, an initially defined absolute margin may no longer be deemed appropriate. In studies of bacterial pneumonia, the US Food and Drug Administration (FDA) considers an absolute margin of 10% acceptable when studying all-cause mortality.^7^ However, as shown in Talbot et al.,^8^ if a certain trial was designed with such an RD margin, but then observed that only 10%–15% of the control patients died, the potential for loss of clinically acceptable efficacy with an absolute 10% margin may be judged too great. A smaller non-inferiority margin may be achieved by changing to an RR or OR scale. Authoritative trials in other disease areas faced similar challenges.^9–11^ A second situation where a scale switch may be considered is when a regression model is used in the analysis phase, for example, for covariate adjustment in sensitivity analyses or in per protocol analyses,^12–14^ or for clustering adjustment in cluster randomized trials. Although attempting to obtain results on the originally planned scale from such regression approaches may be better practice, for example, through marginalization,^15^ sometimes a switch in the analysis scale is made. Finally, when non-inferiority studies are combined in a meta-analysis as stated in Acuna et al.,^16^ converting the scale of the analysis is necessary to allow pooling of study results.

The decision to adjust the scale of the analysis should never be based on the observed comparative (between-arm) outcomes from the study, as this would invalidate results. In line with the potential reasons for switching analysis scale listed above, we assume in the remainder of this article that the decision to change the scale is independent of the between-arm results.

We performed a search for non-inferiority trials with binary outcomes reported in the *New England Journal of Medicine* between 2016 and 2019. Of the 24 randomized controlled trial (RCTs) found, 16 used an RD to specify the non-inferiority margin. Two used RR and six used OR. In nine papers, a different scale from the scale of the main analysis was used to report trial results and/or make an additional analysis. In two papers, the non-inferiority margin was changed related to observing higher or lower than expected event rates.^11,17^ Noticeably in Widmer et al.^17^ paper, non-inferiority could be statistically demonstrated only on the RD scale and not on the RR scale.

Our contribution in this article is threefold. First, we describe sample size consequences when choosing between different scales at the design stage of a trial. We present a new result about how sample size changes when choosing the RR scale compared with the RD scale. Second, we describe changing the scale at a later stage during a trial. We use simulations to provide a comprehensive overview of type I and type II error rates of two ways of mapping the non-inferiority margin. We provide intuition about our results by studying rejection regions. We illustrate the potential impact of the non-inferiority scale in a real trial (i.e. the INES trial^18^ that compared single birth rates in subfertile couples after different fertility treatments). Our results can be used by trialists when choosing the non-inferiority scale at the design stage and when considering performing an analysis on a different scale from the one chosen at the design stage.

## Methods and results

### Choosing between different scales at the design stage

#### Sample size calculation in the INES trial

As a case study, we consider the INES trial that compared two types of in vitro fertilization with intrauterine insemination treatment in couples with unexplained subfertility.^18^ A non-inferiority design was chosen since the in vitro fertilization treatment was expected to prevent more risky twin pregnancies and a slightly lower single birth rate compared with the intrauterine insemination treatment would be acceptable for that reason. The trial was designed anticipating a success rate of 40%, that is, patients achieving a singleton pregnancy within 1 year, in the intrauterine insemination treatment control arm (with either no pregnancy or a non-singleton pregnancy counting as failure). A minimum success rate of 27.5% in the in vitro fertilization treatment arm was considered clinically acceptable. Under the assumption of no real difference between treatments, planning for 80% power and 5% one-sided significance level, the study aimed to exclude an RD of more than –12.5% (27.5% minus 40%), requiring 190 patients per arm. Had the study instead targeted the relative risk, aiming to exclude an RR of 0.69 (27.5% divided by 40%), considerably fewer patients (133 per arm) would have been needed. Strikingly, if the same percentages were formulated as failure rates instead of success rates, that is, the percentage of patients not achieving a singleton pregnancy within 1 year, excluding an RR of 1.21 (72.5% divided by 60%) would require many more patients per arm (235). The fact that the two versions of RR require very different sample sizes may cause confusion to the trial designers. Triggered by this somewhat paradoxical finding, we aimed to systematically examine the effect of the analysis scale in a broad range of design settings. We use some of the design parameters of the INES study as a starting point in our explorations.

#### Notation

We will focus on a two-arm trial with a binary outcome. The data collected in such a trial can be summarized by the success proportions in both arms, estimated from the observed frequencies in the treatment and control arms respectively: ${\hat{p}}_{t}=x_{t}/n_{t}$ , ${\hat{p}}_{c}=x_{c}/n_{c}$ . We will refer to success proportions throughout, but all arguments can also be made using the failure proportions as data summary. We will denote by $p_{c}^{*}$ and $p_{t}^{*}$ the anticipated success proportions during sample size planning (often $p_{c}^{*}=p_{t}^{*}$ ). Let $p_{c}$ and $p_{t}$ denote the ‘true’, unknown success proportions in the control and treatment arm. The treatment effect can be evaluated on four different scales, with $\delta_{\mathrm{RD}}$ the non-inferiority margin on the RD scale, $\delta_{\mathrm{OR}}$ the margin on the OR scale, $\delta_{\mathrm{RR}}$ the margin on the RR scale using the success rates and $\delta_{\mathrm{RR}}^{f}$ the margin on the RR scale using the failure rates, summarized in Supplementary Table S1.

#### Structural comparison of sample sizes

**Figure 1.**
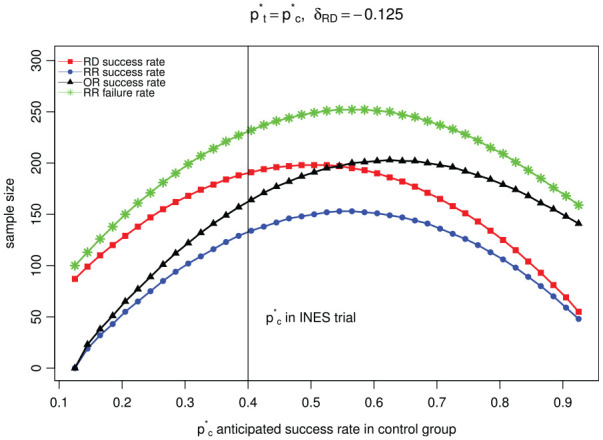
Comparison of sample size when considering different analysis scales at the design stage of the study assuming the boundary proportions for the success rate in treatment group is the same for each scale.

We compared sample size requirements when considering the four analysis scales, mapping the non-inferiority margin in the way illustrated in the INES case study and described more generally in Appendix 1. We rely on the large sample approximation of the (unpooled) Z-test for the sample size calculations (see Supplementary Table S1). The results were quite similar when using other sample size approaches relying on improved approximations.^19^ The difference between the required sample sizes when considering different scales is shown in Figure 1 along a range of control proportions, using a non-inferiority margin of $\delta_{\mathrm{RD}}=-0.125$ , as was used in the INES trial. In Appendix 2, we show similar plots for smaller non-inferiority margins.

The results show that the differences in sample size needed for different scales as described for the INES trial (vertical line in Figure 1) is not an exception. Differences in required sample size when considering different analysis scales can be up to twice as large when comparing the RR using the success rates (bottom line in Figure 1) with the RR using the failure rates (top line in Figure 1).

#### Analytical results

Some of the results we show in Figure 1 can be proven analytically. A comparison of sample size requirement for RD scale and OR scale was given in Rousson and Seifert:^5^ under the assumptions that $p_{c}^{*}=p_{t}^{*}$ and $n_{t}=n_{c}$ , for some given value of $\delta_{\mathrm{OR}}$ , one has that the power when using the RD scale is larger than when using the OR scale as soon as $p_{c}^{*}\geq (1/(1-\delta_{\mathrm{OR}}))+(1/(\ln(\delta_{\mathrm{OR}}))$ .^5^ This result coincides with Figure 1. For $p_{c}^{*}=0.40$ and the minimal acceptable success rate of 0.275 in the treatment arm, as in the INES trial, the $\delta_{\mathrm{OR}}$ is 0.569. According to the result by Rousson and Seifert,^5^ the sample size required for the RD (line with red triangles in Figure 1) should be lower than that needed for the OR (line with black squares in Figure 1) for values of $p_{c}^{*}$ greater than 0.547 and that is exactly where the lines cross.

We here add a proof of the sample size requirements when comparing the RD scale to the RR scale with success proportions. Under the assumptions that $p_{c}^{*}=p_{t}^{*}$ and $n_{c}=n_{t}$ , one has that

$$\begin{array}{c} \frac{n_{\mathrm{RD}}}{n_{\mathrm{RR}}}=\frac{\frac{2{\left(z_{1-\alpha }+z_{1-\beta }\right)}^{2}p_{c}^{*}\left(1-p_{c}^{*}\right)}{{\left(\delta_{\mathrm{RD}}\right)}^{2}}}{\frac{2{\left(z_{1-\alpha }+z_{1-\beta }\right)}^{2}\frac{\left(1-p_{c}^{*}\right)}{p_{c}^{*}}}{{\left(\ln\left(\delta_{\mathrm{RR}}\right)\right)}^{2}}}\\ \;=\frac{{\left(p_{c}^{*}\right)}^{2}}{{\left(\delta_{\mathrm{RD}}\right)}^{2}}\left(\ln\left(p_{c}^{*}+\delta_{\mathrm{RD}}\right)-\;\ln\left(p_{c}^{*}\right)\right){\;}^{2}\\ \;=\frac{1}{{\left(\frac{\delta_{\mathrm{RD}}}{p_{c}^{*}}\right)}^{2}}{\left(\ln\left(1+\frac{\delta_{\mathrm{RD}}}{p_{c}^{*}}\right)\right)}^{2}\\ \;\in \left(1,+\infty \right) \end{array}$$

where the last relation holds because $\left(\delta_{\mathrm{RD}}/p_{c}^{*})\in (-1,0\right)$ in a non-inferiority trial. This shows that the sample size needed using the RR scale with success proportions (blue dotted line at the bottom in Figure 1) is always lower than the sample size needed using the RD scale (line with red squares in Figure 1).

### Changing the scale at the analysis stage

#### Re-analysis of the INES trial

Based on observed single pregnancy rates in the 602 study participants (52% for the 201 patients allocated to the in vitro fertilization with single embryo transfer treatment, 43% for the 194 patients allocated to the in vitro fertilization in a modified natural cycle treatment and 47% for the 207 patients allocated to the intrauterine insemination treatment respectively), the study investigators concluded that both the in vitro fertilization with single embryo transfer treatment and the in vitro fertilization in a modified natural cycle treatment were non-inferior to the intrauterine insemination treatment.^20^ As pointed out in Van Geloven,^21^ the trial reported results on the RR scale, whereas the sample size calculation had been based on the RD scale. A recalculation of the main study results using different scales shows that the trial could have reached a different conclusion had it been analysed on the RD scale (Table 1, Van Geloven^21^). As shown in Table 1, regardless of the scale used to report the results, the in vitro fertilization with single embryo transfer treatment can consistently be concluded to be non-inferior to the intrauterine insemination treatment. However, if one uses different scales to report the results of the in vitro fertilization in a modified natural cycle treatment versus the intrauterine insemination treatment, the conclusions are inconsistent. Specifically, when the OR or the RR with success rate is used, one can draw the conclusion that the in vitro fertilization in a modified natural cycle treatment is non-inferior to the intrauterine insemination treatment (using a 2-sided alpha of 0.05). On the contrary, one cannot conclude that the in vitro fertilization in a modified natural cycle treatment is non-inferior to the intrauterine insemination treatment when the RD or the RR with failure rate is used. Particularly, the contradictory conclusions drawn by using the RR scale with success rate and failure rate, respectively, may pose a dilemma for trialists as to whether non-inferiority should be accepted.

**Table 1.** Recalculation of the INES trial main study results that compared two types of in vitro fertilization, that is, the IVF-SET treatment and the IVF-MNC treatment, to the IUI treatment.

Comparison	Margin type	Estimate	95% confidence interval	p-value for NI	Conclusion
IVF-SET vs IUI	RD	5%	(–5% to 14%)	<0.001	NI met
	RR success rate	1.11	(0.91 to 1.35)	<0.001	NI met
	OR	1.22	(0.82 to 1.79)	<0.001	NI met
	RR failure rate	0.91	(0.75 to 1.10)	0.003	NI met
IVF-MNC vs IUI	RD	–4%	(–14% to 6%)	0.090	NI failed
	RR success rate	0.91	(0.73 to 1.13)	0.012	NI met
	OR	0.85	(0.57 to 1.26)	0.048	NI met
	RR failure rate	1.08	(0.90 to 1.29)	0.195	NI failed

NI: non-inferiority; IVF-SET: in vitro fertilization with single embryo transfer; IUI: intrauterine insemination; RD: risk difference; RR: risk ratio; OR: odds ratio; IVF-MNC: in vitro fertilization in a modified natural cycle. Confidence intervals were calculated by score method. The RD margin is –12.5% (27.5% – 40%), the RR margin with success rate is 0.69 (27.5% / 40%), the OR margin is 0.57 ((27.5% / 72.5%) / (40% / 60%) and the RR margin with failure rate is 1.21 (72.5% / 60%). two times one-sided p-values for NI are presented.

#### Structural comparison of type I and type II error rates

When a change in the scale is made after the trial has commenced, sample size calculation has already been performed and is no longer of main interest. Therefore, for such switches, we examined power, that is, one minus type II error rate, and type I error rate, based on simulations assuming a fixed sample size. We consider two ways of mapping the non-inferiority margin to the new scale: either based on the anticipated control proportion (similar to what was done in the INES trial and in Widmer et al.)^17^ or based on the observed control proportion. In the latter case, again starting with an RD, this means that the non-inferiority margin is added to the observed success proportion in the control arm ${\hat{p}}_{c}$ to come to the minimum allowed success proportion in the treatment arm, $p_{t}^{\inf,2}$ . By comparing $p_{t}^{\inf,2}$ and ${\hat{p}}_{c}$ , the new margins on the RR scale and the OR scale can be obtained (see Supplementary Table S2).

##### Comparison of power

We simulated the success proportions of 100,000 trials similar in setup to the INES trial (sample size 190, one-sided α=0.05 , $\delta_{\mathrm{RD}}=-0.125$ ) using binomial distributions according to the alternative hypothesis with anticipated proportions $p_{t}^{*}=p_{c}^{*}=0.40$ . Power was calculated as the proportion of trials in which $H_{0}$ was correctly rejected.

**Figure 2.**
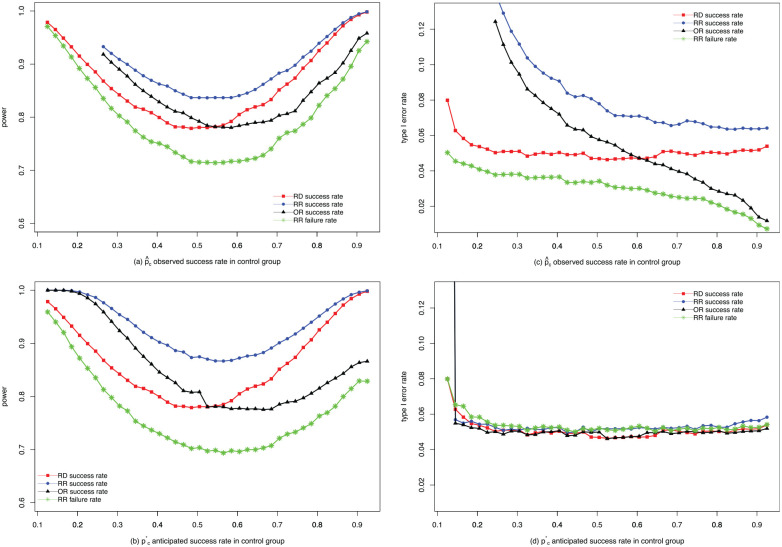
Comparisons of power and type I error rate. (a) comparison of power when mapping using the observed control proportion; (b) comparison of power when mapping using the anticipated control proportion; (c) comparison of type I error rate when mapping using the observed control proportion; (d) comparison of type I error rate when mapping using the anticipated control proportion.

Results are presented in Figure 2(a) and (b). Under these settings, the power for the RR scale using the success rate is always the highest (top blue dotted line), while the power for the RR scale using the failure rate is always the lowest (bottom green line with stars). In addition, the power on the RD scale and the OR scale lies between them, crossing at some point. This shows that power increases when switching from the RD scale to the RR scale, both when using the anticipated and when using the observed control success proportion during mapping of the non-inferiority margin. The differences in power when switching using the anticipated control proportion are larger than when using observed control proportion according to our simulations.

##### Comparison of type I error rate

For type I error rate, we simulated 100,000 trials with similar design with success proportions specified by the null hypothesis $p_{t}=p_{c}+\delta_{\mathrm{RD}}$ , where the trial is originally designed on the RD scale. Type I error rate was calculated as the proportion of trials that incorrectly rejected $H_{0}$ . When switching using the anticipated control proportion, the type I error rates on different scales were close to each other, wiggling between 0.05 and 0.055 in most cases (Figure 2(d)). When the non-inferiority margin is mapped using the observed control proportion, it can be seen that the type I error rate on the RR scale with success proportion is unacceptably high on all occasions, whereas the type I error rate on the RR scale using the failure proportion is too low. Moreover, the type I error rates on the RD scale and the OR scale are in-between and cross at around 60% observed control success rate. One can infer that the adaptive nature of this way of mapping fails to preserve type I error rate and therefore should not be advised.

##### Understanding the differences in type I and type II error rates through rejection regions

The differences in type I and type II error rates that we found can be understood by looking at rejection regions. We show these as region plots of the results of the simulated trials in Appendix 3 for switching using anticipated proportions and in Appendix 4 for switching using observed proportions. The figures make clear that analyses on different scales will agree on rejecting the null hypothesis or not in trials where the observed rates ( ${\hat{p}}_{c}$ and ${\hat{p}}_{t}$ ) are close to the anticipated proportions ( $p_{c}^{*}$ and $p_{t}^{*}$ ). However, because of chance variations, some of the trials will have a larger than expected success rate in the control arm and/or a lower than expected success rate in the treatment arm. In such trials, analyses on different scales will reach different conclusions concerning rejecting. We present rejection regions based on simulations for other designs, for example, designs with unequal anticipated success rates in Appendix 5.

## Discussion

We showed that differences in sample size requirements can occur when considering different analysis scales at the design stage of a non-inferiority trial. The main impact of changing the scale at the analysis stage using anticipated proportions for mapping the non-inferiority margin is on power. By studying rejection regions, we made clear that these results are not due to different inference (e.g. larger standard errors), but instead are caused by the fact that the choice of a particular scale plus non-inferiority margin defines a full rejection region. The regions of two scales only coincide when observed rates are close to anticipated ones, but will differ when the observed proportions deviate from expectations. Moreover, even if we use the same scale to design and analyse a non-inferiority trial, using the RR scale with success rate and failure rate may lead to contradictory conclusions. This raised the question of the appropriateness of using RR for non-inferiority trials. Mapping the non-inferiority margin relative to the observed proportion in the control arm introduces problems as the evaluation criteria become too dependent on random low or high observed proportions. This is reflected in strongly in- or deflated type I error rates and matches the adaptive nature of the method. In general, we advise against such data-dependent mapping. If it is considered, then a correction for type I error rate inflation must be used. Some advice for simulation-based correction methods are given in Quartagno et al.^22^

The issues we describe are particularly important for non-inferiority trials since changing the analysis scale requires redefining the non-inferiority margin. In superiority trials, the neutral comparison values (zero for the RD and one for the RR or OR) do not change when switching the analysis scale such that no large differences between scales are expected.

Analysing a trial in a different way from designed is considered bad practice in general. Whenever possible, we advise keeping the assessment of non-inferiority on the originally planned analysis scale. If the analysis (e.g. a regression model) is performed on another scale, marginalization techniques can be used to report end results on the original scale. But as explained in the introduction section, changes may not be avoidable at times. A change in the analysis scale should not be made lightly. Changing the scale means that trialists commit to a different boundary region of what they accept as clinically acceptable difference. It means that they realized that the original scale used was not correct. In fact, the simulation results that we described only hold if the hypotheses are formulated according to the new scale. For example, if the null hypothesis is formulated using the old scale and the control event rate is different from expected, then the type I error rate will no longer be maintained when changing the margin according to anticipated control rate. Our results should also not be read as encouragement to change the scale of a trial to gain power. Increased (or decreased) power can be a consequence of changing the scale but it should never be the reason for changing as the clinical judgement on what is an acceptable margin cannot be overruled by statistical arguments. Switching needs to be done in an unbiased way, meaning that any new margin implied by a different scale has to be justified clinically and must reflect new insights into the study design based on outcome blinded analyses. This process must be carefully and transparently described to avoid optimistic interpretation of data. It is helpful to mention consequences for power although they must not inform the switch.

To avoid having to change the scale, we recommend to consider at the design stage all clinical and trial size implications, including scenarios where the event rates are higher or lower than expected and discuss whether the chosen margin would still suffice in such a situation. If a switch is unavoidable, we strongly recommend against switching based on the final observed control event rate, but to use the anticipated rate instead. Anticipated rates may potentially be updated based on blinded interim analysis but we did not study this in detail. Quartagno et al.^22^ recently proposed a more flexible way of defining the non-inferiority region, recommending the use of the arc-sine scale because of its power-stabilizing properties.

We hope to have made clear that changing the scale in a non-inferiority trial is not without consequences and trialists should consider the impact on type I and type II error rates before such a switch is made.

## Supplemental Material

sj-pdf-1-ctj-10.1177_17407745211053790 – Supplemental material for Choosing and changing the analysis scale in non-inferiority trials with a binary outcome Click here for additional data file. Supplemental material, sj-pdf-1-ctj-10.1177_17407745211053790 for Choosing and changing the analysis scale in non-inferiority trials with a binary outcome by Zhong Li, Matteo Quartagno, Stefan Böhringer and Nan van Geloven in Clinical Trials

## Appendix 1

### Comparison of sample size when switching using the anticipated control proportion with smaller margins

In Supplementary Figures S1–S3, with one-sided α=0.05 and power = 0.80, we present the results of comparison of sample size when switching using the anticipated control proportion, given $\delta_{\mathrm{RD}}=-0.10$ , $\delta_{\mathrm{RD}}=-0.05$ and $\delta_{\mathrm{RD}}=-0.01$ , respectively.

## Appendix 2

### Illustrations of simulated rejection regions when switching using the anticipated control proportion and p^*^_t_ = p^*^_c_

As shown in Supplementary Figure S4, we demonstrate the simulated rejection regions for power when switching using the anticipated control proportion (with 100,000 simulations, n=190 , $p_{c}^{*}=p_{t}^{*}=0.40$ and $\delta_{\mathrm{RD}}=-0.125$ ). Specifically, when the trial is designed on the risk difference (RD) scale, regardless of the scales used to the report the trial results, all trial outcomes summarized by ( ${\overset{\cdot }{p}}_{t},\;{\overset{\cdot }{p}}_{c}$ ) located in the area composed of green solid circles conclude that the treatment is non-inferior to the control (rejection of the null hypothesis of non-inferiority). For trials with outcomes ( ${\overset{\cdot }{p}}_{t},\;{\overset{\cdot }{p}}_{c}$ ) located in the area composed of black circles analyses on all scales, one cannot conclude that the treatment is non-inferior to the control (no rejection of the null hypothesis). However, for trials with outcomes ( ${\overset{\cdot }{p}}_{t},\;{\overset{\cdot }{p}}_{c}$ ) located in the area composed of blue solid circles, one can conclude that the treatment is non-inferior to the control when using the risk ratio (RR) or odds ratio (OR) scale to analyse the trial results, while one cannot draw this conclusion if the RD scale is used. Similarly, for trials with ( ${\overset{\cdot }{p}}_{t},\;{\overset{\cdot }{p}}_{c}$ ) located in the area composed of red solid circles, one can conclude that the treatment is non-inferior to the control when using the RR scale to analyse the trial results, while one cannot draw this conclusion if the RD or OR scale is used. Therefore, this figure intuitively explains why power changes when different scales are used to analyse the trial results based on anticipated rate.

Considering that a type I error is the rejection of a true null hypothesis, the same method was used for Supplementary Figure S5, which intuitively explains why type I error rate changes when different scales are used to analyse the trial results and switching using the anticipated control proportion.

## Appendix 3

### Illustrations of simulated rejection regions when switching using the observed control proportion

As shown in Supplementary Figure S6, we demonstrate the simulated rejection regions for power when switching using the observed control proportion (with 100,000 simulations, n=190 , $p_{c}^{*}=p_{t}^{*}=0.40$ and $\delta_{\mathrm{RD}}=-0.125$ ). Regarding the type I error rate, the same analysis method was used for Supplementary Figure S7. Conclusions similar to Appendix 3 can be drawn.

## Appendix 4

### Illustrations of simulated rejection regions when using anticipated rate and p^*^_t_≠ p^*^_c_

As shown in Supplementary Figures S8 and S9, we demonstrate the simulated rejection regions for power when switching using the anticipated control proportion and $p_{t}^{*}\neq p_{c}^{*}$ (with 100,000 simulations, n=190 , $p_{c}^{*}=0.40$ , $\delta_{\mathrm{RD}}=-0.125$ , $p_{t}^{*}=p_{c}^{*}+0.05$ or $p_{t}^{*}=p_{c}^{*}-0.05$ , respectively). Conclusions similar to Appendix 3 can also be drawn.

## Appendix 5

### Supplementary tables

In the sample size formulas presented in Supplementary Table S1, it is assumed that for each analysis scale, an independent non-inferiority margin was chosen. When considering different analysis scales at the design stage of a study, it is not uncommon that researchers will first choose the ‘boundary success rate’ that is still allowed in the treatment arm and then translate it to non-inferiority margins on different scales as illustrated in the INES case study.

Sample size calculations use an anticipated success proportion in the control arm $p_{c}^{*}$ . Now suppose that the margin of non-inferiority is initially defined on the risk difference scale, say $\delta_{\mathrm{RD}}$ . Note that $\delta_{\mathrm{RD}}$ is negative when using the success proportion. Under the null hypothesis of inferiority, this results in the following boundary success proportion in the treatment arm $p_{t}^{\inf,1}=p_{c}^{*}+\delta_{\mathrm{RD}}$ . Based on the two proportions $p_{c}^{*}$ and $p_{t}^{\inf,1}$ , margins on the other analyses scales may be chosen alternatively. Supplementary Table S2 lists the margins when mapping non-inferiority margins in this way and in a second way that uses observed proportions.
